# Arc is a flexible modular protein capable of reversible self-oligomerization

**DOI:** 10.1042/BJ20141446

**Published:** 2015-05-05

**Authors:** Craig Myrum, Anne Baumann, Helene J. Bustad, Marte Innselset Flydal, Vincent Mariaule, Sara Alvira, Jorge Cuéllar, Jan Haavik, Jonathan Soulé, José Maria Valpuesta, José Antonio Márquez, Aurora Martinez, Clive R. Bramham

**Affiliations:** *Dr Einar Martens' Research Group for Biological Psychiatry, Center for Medical Genetics and Molecular Medicine, Haukeland University Hospital, 5009 Bergen, Norway; †Department of Biomedicine, University of Bergen, Jonas Lies vei 91, N-5009 Bergen, Norway; ‡KG Jebsen Center for Research on Neuropsychiatric Disorders, University of Bergen, Jonas Lies vei 91, N-5009 Bergen, Norway; §European Molecular Biology Laboratory, Grenoble Outstation, 72 rue Jules Horowitz, BP181, 38042 Grenoble Cedex 9, France; ║Centro Nacional de Biotecnología (CNB-CSIC), Darwin 3, 28049 Madrid, Spain; ¶Unit of Virus Host-Cell Interactions, University Grenoble Alpes-EMBL-CNRS, 6 rue Jules Horowitz, 38042 Grenoble, France

**Keywords:** Arc, biophysical characterization, oligomerization, presenilin-1, protein purification, protein stability, synaptic plasticity, Aβ, amyloid β-peptide, AMPA, α-amino-3-hydroxy-5-methyl isoxazole-4-propionic acid, APP, amyloid precursor protein, Arc, activity-regulated cytoskeleton-associated protein, *D*_h_, hydrodynamic diameter, DLS, dynamic light scattering, DSC, differential scanning calorimetry, DSF, differential scanning fluorimetry, F-actin, filamentous actin, hArc, human Arc, HCA, hydrophobic cluster analysis, LTD, long-term depression, LTP, long-term potentiation, MBP, maltose-binding protein, Ni-NTA, Ni^2+^-nitrilotriacetate, PS1, presenilin-1, RT, room temperature, RU, response units, SPR, surface plasmon resonance, TBST, Tris-buffered saline/0.1% Tween 20, TEV, tobacco etch virus, Trx, thioredoxin, WT, wild-type

## Abstract

The immediate early gene product Arc (activity-regulated cytoskeleton-associated protein) is posited as a master regulator of long-term synaptic plasticity and memory. However, the physicochemical and structural properties of Arc have not been elucidated. In the present study, we expressed and purified recombinant human Arc (hArc) and performed the first biochemical and biophysical analysis of hArc's structure and stability. Limited proteolysis assays and MS analysis indicate that hArc has two major domains on either side of a central more disordered linker region, consistent with *in silico* structure predictions. hArc's secondary structure was estimated using CD, and stability was analysed by CD-monitored thermal denaturation and differential scanning fluorimetry (DSF). Oligomerization states under different conditions were studied by dynamic light scattering (DLS) and visualized by AFM and EM. Biophysical analyses show that hArc is a modular protein with defined secondary structure and loose tertiary structure. hArc appears to be pyramid-shaped as a monomer and is capable of reversible self-association, forming large soluble oligomers. The N-terminal domain of hArc is highly basic, which may promote interaction with cytoskeletal structures or other polyanionic surfaces, whereas the C-terminal domain is acidic and stabilized by ionic conditions that promote oligomerization. Upon binding of presenilin-1 (PS1) peptide, hArc undergoes a large structural change. A non-synonymous genetic variant of hArc (V231G) showed properties similar to the wild-type (WT) protein. We conclude that hArc is a flexible multi-domain protein that exists in monomeric and oligomeric forms, compatible with a diverse, hub-like role in plasticity-related processes.

## Abstract 2

A series of complementary biochemical and biophysical analyses revealed that the human activity-regulated cytoskeleton-associated protein (hArc) protein has a modular structure with two domains, is monomeric but also forms oligomers, which posits Arc to be a flexible hub protein.

## INTRODUCTION

Storage of information in the brain is thought to require the plasticity of neural circuits. Stable forms of synaptic plasticity have long been known to depend on neuronal activity-induced protein synthesis [1,2]. The immediate early gene product, activity-regulated cytoskeleton-associated protein (Arc/Arg3.1), has emerged as a key protein in memory formation and diverse types of synaptic plasticity including long-term potentiation (LTP), long-term depression (LTD) and homoeostatic synaptic scaling [3–6]

In the first papers describing Arc, the authors reported 20% sequence similarity between a C-terminal region of Arc (rat amino acids 228–380 of 396) and two α-spectrin repeats [7,8]. Spectrin repeats are diverse and co-ordinate cytoskeletal interactions and can serve as a ‘switchboard’ for interactions with multiple proteins [9]. Arc in fact binds to a nuclear spectrin isoform, βSpIVΣ5, in promyelocytic leukaemia (PML) bodies in the nucleus [10]. A recent study shows that nuclear Arc functions to decrease transcription of the AMPA (α-amino-3-hydroxy-5-methyl isoxazole-4-propionic acid)-type glutamate receptor subunit GluA1, resulting in a homoeostatic downscaling of excitatory synaptic transmission [11]. At synapses, Arc interacts with components of the clathrin-mediated endocytosis machinery, endophilin-3 and dynamin-2, to promote internalization of AMPA receptors and LTD [12,13]. Arc also recruits presenilin-1 (PS1) and the γ-secretase complex to endosomes, resulting in enhanced processing of amyloid precursor protein (APP) and Notch1 [14,15]. Arc protein synthesis is critical in the consolidation phase of LTP [16–19], a process that also requires actin cytoskeletal remodelling in dendritic spines [20,21]. During LTP, Arc promotes filamentous actin (F-actin) stabilization and phosphorylation of the actin-binding protein cofilin [17]. Furthermore, Arc has been suggested to interact with newly polymerized microtubules [22].

Taken together, evidence suggests that Arc interacts with several distinct proteins to regulate multiple cellular processes. Despite 20 years of research on Arc, little is known about the basic properties of the protein with regard to its structure and stability. Such information is essential if we are to gain a molecular understanding of Arc function in synaptic plasticity and cognition. In the present work, we have expressed and purified recombinant human Arc (hArc) and we have characterized the protein by using a number of complementary biochemical, biophysical and microscopy analyses. The results indicate that hArc is a flexible protein consisting of two major domains on either side of a central, mostly unstructured, hinge region. Furthermore, we show that hArc is monomeric and capable of reversible self-oligomerization. The N-terminal domain is highly basic and stabilized by heparin, whereas the C-terminal domain is acidic and stabilized by increasing salt concentration. The stabilization of the Arc C-terminal domain is associated with oligomerization. Furthermore, we show that a peptide corresponding to the N-terminal cytoplasmic region of the hArc partner PS1 [14] binds to hArc and rearranges its conformation. These findings indicate that hArc is a flexible multi-domain protein capable of reversible self-oligomerization.

The 1000 Genomes Project [23] uncovered a single non-synonymous *ARC* variant, which results in an amino acid substitution of glycine for valine at position 231 (rs201562490). The V231G polymorphism is present in around 2–4.6% of the population and to the best of our knowledge no phenotypic characterization has been reported. In the present study, we demonstrate that V231G hArc has similar structural properties as the wild-type (WT) hArc protein.

## MATERIALS AND METHODS

### *In silico* Arc analysis

A multiple sequence alignment was performed using Clustal Omega software [24] and viewed in Jalview 2 [25]. Consensus was calculated based on an algorithm analysing the physicochemical properties of sets of amino acids in the multiple sequence alignment [26]. MeDor, a meta-predictor of protein structure [27], was used to analyse the primary hArc sequence. MeDor outputs graphical displays of various prediction methods including a hydrophobic cluster analysis (HCA) plot [28]. Horizontal clusters indicate α-helices and vertical shapes mainly correspond to β-strands. Publicly available structure models of hArc were obtained from MODBASE [29], which contains theoretically calculated models based on homology. Reliability was measured using five different methods with the following thresholds; MPQS (ModPipe Quality Score): ≥1.1, TSVMod NO35 [estimated native overlap at 3.5 Å (1 Å=0.1 nm)]: ≥40%, GA341: ≥0.7, *E*-value: <0.0001, zDOPE: <0.

### Preparation of recombinant human Arc

The Arc coding sequence of hArc was amplified by PCR from a cDNA library using the primers 5′-GCTT-CCATGGAGCTGGACCACC-3′ and 5′-GCTTGGTACC-CTACTCGGGCTGG-3′ (the NcoI and Acc65I restriction sites are underlined). The PCR product was ligated into pETMBP_1a [maltose-binding protein (MBP)], pETZZ_1a, pETTrx_1a [thioredoxin (Trx)] and pETGST_1a [30]. The resulting constructs were transformed into One Shot® TOP10 Chemically Competent Cells (Invitrogen). Plasmids were sequenced before being transformed into BL21-CodonPlus Competent Cells (Agilent) for protein expression.

A pilot expression and purification study was performed to test expression from each of the plasmids in 2 ml of auto-induction medium [31]. Cultures were incubated with vigorous shaking at 27°C overnight, harvested, resuspended in 10 ml/g lysis buffer (20 ml Tris/HCl, pH 7.4, 10 mM imidazole, 150 mM NaCl, 0.2% NP-40, 10 mM mercaptoethanol and 10% glycerol), sonicated and centrifuged again. The supernatant was pressed through 0.45 μm pore size filters and loaded on to columns of 30–50 μl of Ni-NTA (Ni^2+^-nitrilotriacetate)–agarose resin (Qiagen). The resin was sequentially washed with the following buffers: (1) lysis buffer, (2) lysis buffer without NP-40, (3) wash number 2 with 1 M NaCl, and (4) wash number 2 with 40 mM imidazole. Bound protein was eluted with 330 mM imidazole. Most of the experiments in the present work were performed using hArc cleaved from the His–ZZ–hArc fusion protein, and for upscaled pET ZZ_1a-hArc cultures, ~4 ml of Ni–NTA–agarose (Qiagen) was used.

The His–ZZ expression tag was completely cleaved off by adding 1:100 (w/w ratio) His-tagged TEV (tobacco etch virus) protease directly to the dilute eluate from the affinity column and left to incubate for 12 h at 4°C. After incubation, the volume was reduced to 2.5 ml using 30 kDa cut-off centrifuge filters (Vivaspin) and buffer-exchanged to 20 mM Tris/HCl, pH 7.4, 150 mM NaCl and 10 mM mercaptoethanol, using PD-10 Desalting Columns (GE Healthcare) to remove imidazole. The eluate was loaded on columns of 30–50 μl of Ni-NTA–agarose to remove the His–ZZ tag and His-tagged TEV. The flow-through (containing pure hArc protein, either WT or V231G hArc variant) was collected. The GST–hArc fusion protein, purified by glutathione-Sepharose 4B (GE Healthcare Life Sciences), was used when indicated.

Site-directed mutagenesis of pET ZZ_1a-hArc was performed using the QuikChange SDM kit (Stratagene) according to the manufacturer's protocol (primers: 5′-TACTTGC-GGCAGGGGGGCGGC TCTGAGG-3′ and 5′-CATGAACGC-CGTCCCCCC GCCGAGACTC-3′; mutated bases are underlined). Mutagenesis was confirmed by DNA sequencing. The protein was then purified as described above.

Buffer exchange to the conditions specific in each experiment was performed by running the samples through Zeba Spin Desalting Columns (Thermo Scientific) using the manufacturer's protocol.

### Cell culture

Human SH-SY5Y cells (A.T.C.C.) were grown in Dulbecco's modified Eagle medium (DMEM; Sigma) supplemented with 10% FBS, penicillin/streptomycin and L-glutamine. Cells were lysed in PBS containing 0.1% Triton-X100, 1 mM PMSF and Roche Complete Protease Inhibitor Cocktail.

### SDS/PAGE and immunoblot analysis

Protein (40 μg) was separated on SDS/PAGE (10% gel) and transferred on to a nitrocellulose membrane (Hybond-C). Membranes were blocked for 1 h at room temperature (RT) in TBST (Tris-buffered saline/0.1% Tween 20) and 3% non-fat dried milk. Arc C-7 primary antibody (Santa Cruz Biotechnology) was diluted in blocking buffer containing TBST and 5% BSA and applied on membranes overnight at 4°C with constant shaking. Following three washes with TBST, blots were incubated for 1 h at RT in horseradish peroxidase-conjugated secondary antibody diluted in TBST. Blots were then visualized using ECL (Pierce, ECL Western Blotting Substrate).

### Limited proteolysis

Aliquots of 13 μg of hArc were individually digested with either trypsin or chymotrypsin. Both proteases were prepared using 25 mM sodium Hepes (pH 7.4) buffer. Proteolytic digestions were performed on ice with protease/hArc ratios of 1:100, 1:1000, 1:10000 and 1:100000. The reactions were stopped by adding 2 μl of protease inhibitor solution (1 Roche Complete Protease Inhibitor Cocktail tablet in 500 μl of 25 mM sodium Hepes, pH 7.4) at different time points. Ten microlitres of the reaction products was loaded on a SDS/PAGE (15% gel). Chymotrypsin was chosen for further analyses at a protease/hArc ratio of 1:100. The process was repeated over a time course of 120 min and the appearance of fragments was visualized by SDS/PAGE. MS was performed on both chymotrypsin and trypsin digestion fragments.

### MS

Digestion bands were cut from the gel with a clean scalpel. Gel pieces were then cut into 1-mm cubes followed by in-gel hydrolysis. The gel pieces were washed, reduced using DTT and alkylated with iodacetamide. The dehydrated gel pieces were transferred to glass tubes (300 μl) and 20–30 μl of 3 M HCl was added and the tubes were microwaved for 10 min at 900 W. The supernatant was removed and desalted directly on Oasis HLB Elution Plate (Waters). Samples were eluted in 50 μl, dried in a speed vacuum centrifuge and dissolved in 10 μl of reconstitution buffer (96:4 water/acetonitrile and 0.1% formic acid) and analysed by LC–MS/MS. Here, peptides were separated using the nanoAcquity UPLC system (Waters) fitted with a trapping (nanoAcquity Symmetry C_18_, 5 μm, 180 μm × 20 mm) and analytical column (nanoAcquity BEH C_18_, 1.7 μm, 75 μm × 200 mm) coupled directly to an linear trap quadropole (LTQ) Orbitrap Velos (Thermo Fisher Scientific) with a Proxeon nanospray source. Solvent A was water+0.1% formic acid and solvent B was acetonitrile+0.1% formic acid. The samples (8 μl) were loaded with a constant flow of solvent A at 5 μl/min on to the trapping column. Peptides were eluted via the analytical column at a constant flow of 0.3 μl/min. The peptides were introduced into the mass spectrometer (Orbitrap Velos Pro, Thermo) via a Pico-Tip Emitter 360 μm OD (outer diameter) × 20 μm ID (inner diameter); 10 μm tip (New Objective) and a spray of 2.2 kV was applied. Full scan MS spectra with mass ranges of 300–1700 *m*/*z* were acquired in the fourier transform (FT) profile mode with resolution of 30000. The most intense ions (up to 15) from the full scan MS were selected for sequencing in the LTQ. MS/MS data were acquired in centroid mode. Only multiply charged precursor ions (2+, 3+, 4+) were selected for MS/MS.

Data analysis was performed using MaxQuant Software (version 1.0.13.13) for filtering the data and creating .mgf files, needed for searching in MASCOT version 2.2.03 (Matrix Science). The data were searched against a species-specific (*Homo sapiens*) Uniprot database with a list of common contaminants appended. The data were searched with the following modifications: carbamidomethyl (C; fixed) and oxidation (M; variable). Termini were postulated based on peptide ladders of increasing amino acid length, either all starting or all ending at the same residue (for N- and C-termini respectively).

### CD

Far-UV and thermal denaturation CD measurements were performed with a J-810 Jasco spectropolarimeter equipped with a CDF-426S Peltier element for temperature control using a quartz cell with a path length of 1 mm. The buffer containing purified hArc was exchanged using Zeba Spin Desalting Columns with 10 mM potassium phosphate with various pHs, potassium fluoride (KF) concentrations or additives such as heparin (Abbott Laboratories) and calcium [added as Ca(NO_3_)_2_], as indicated. The final protein concentration was 4.4 μM or 4.0 μM when testing in the presence of 10 μM of a 40-residue peptide corresponding to the cytoplasmic N-terminal region of PS1 (from Tag Copenhagen). Spectra were acquired in the 185–260 nm range at a scan rate of 50 nm/min at 20°C. Two scans were accumulated for each spectrum and three spectra were buffer-subtracted and averaged. Thermal denaturation profiles were obtained by recording the ellipticity at 222 nm as a function of temperature in the range 4–100°C with a scan rate of 2°C/min. The far-UV CD spectra and thermal denaturation profiles were smoothed using a negative exponential algorithm with a sample proportion=0.05 and polynomial degree=1. Circular dichroism by neural networks (CDNN) [32] was used to estimate the secondary structure content.

### Differential scanning fluorimetry

Samples with 2 μM hArc were prepared in 20 mM sodium Hepes, pH 7.0, with 5× SYPRO Orange (Sigma–Aldrich) with or without 150 mM KF and added to wells in a 96-well plate with decreasing concentrations of heparin (25 μg/ml to 0 μg/ml). The plates were loaded into a Light Cycler 480 (Roche Applied Science) and heated from 20°C to 90°C at a scan rate of 2°C/min. Unfolding was monitored by following the increase in SYPRO Orange fluorescence (*λ*_ex_=465 nm, *λ*_em_=610 nm). Half-denaturation temperature values (*T*_m_ values) were obtained from the maximum first derivative of the raw data.

### Dynamic light scattering

Dynamic light scattering (DLS) was performed on a Malvern Zetasizer Nano ZS with a HeNe laser at 633 nm. Temperature scans and size measurements were carried out at a fixed scattering angle of 173° (back scatter). Temperature scans were run from 4°C to 90°C at a rate of 2°C/min. Purified protein preparations were diluted to 1 mg/ml (22 μM) in the indicated buffers and additives. Data analysis was performed on intensity and volume size distribution curves and the molecular mass and Z-average size was calculated using Malvern DTS software. The intensity size distribution, the first order result, is weighted according to scattered intensity of each particle fraction whereas the volume size distribution represents the relative proportion based on the mass or volume, which is derived from the intensity size distribution via the Mie theory. The Z-average provides a reliable measure of the mean size of the particle size distribution [33].

### EM

Samples of hArc under different conditions (water; 20 mM sodium Hepes, pH 7.0; 20 mM sodium Hepes, pH 7.0, with 125 mM KCl) were applied to glow-discharged carbon grids at RT for 3 min, washed and then stained for 2 min with 2% (w/v) uranyl acetate. Micrographs were recorded on a JEOL 1200EX-II electron microscope operated at 100 kV on Kodak SO-163 film.

### AFM imaging

Samples were prepared by spreading 50 μl of hArc (11.25 μg/ml; 0.25 μM) in 10 mM potassium phosphate, pH 7.4, on a freshly cleaved mica surface (diameter 1.2 cm), incubated for 10 min and gently washed with milliQ distilled water. When dried, AFM imaging was carried out in air at RT. All images were generated with the tapping mode (AC mode) on an MFP-3D-Bio™ atomic force microscope (Asylum research, an Oxford Instruments company) using silicon cantilevers, AC240, from Olympus with a typical spring constant of 2 N/m. Images were captured with a resolution of 256 pixels × 256 pixels and the scan rate was adjusted for each sample to a value between 0.5 and 1 Hz. At least three regions of the sample surface were investigated to confirm homogeneity. All images were processed using IGOR PRO (Wavemetrics).

### Surface plasmon resonance

Surface plasmon resonance (SPR) analyses were carried out at 25°C using a Biacore 3000 instrument (GE Healthcare) with HBS-EP (10 mM sodium Hepes, pH 7.4, 150 mM NaCl, 3 mM EDTA and 0.005% surfactant P20) as running buffer. Anti-GST antibody (GST Capture Kit, GE Healthcare) was immobilized by amine coupling to a level of 9210 response units (RU) on to a CM5 sensor chip (GE Healthcare) according to the manufacturer's instructions. GST–hArc fusion protein (3 μg/ml, 66 nM) was reversibly captured at a flow rate of 30 μl/min and a 3-min injection to a level of 280–320 RU. Interaction with PS1 was tested by injecting a series of dilutions of the peptide (100–400 μM) over the GST–hArc surfaces at a flow rate of 30 μl/min and a 2-min injection. Peptide A (EQLTKCEVFRELKDLKGY; obtained from CPC Scientific Inc.), corresponding to the N-terminal region of α-lactalbumin, was treated in the same way as PS1 and used as a negative control. Regeneration of anti-GST antibody surfaces was accomplished with a 30 μl/min injection of 10 mM glycine/HCl, pH 2.1, for 2 min. All data generated were subtracted from the reference surface (no anti-GST antibody immobilized). BIAevaluation software (Version 3.2; GE Healthcare) was used for analysis of the sensorgrams. Dissociation constants (*K*_d_) were calculated by fitting the sensorgrams to a 1:1 Langmuir binding model via non-linear regression analysis.

## RESULTS

### Prediction of hArc structure

**Figure 1:**
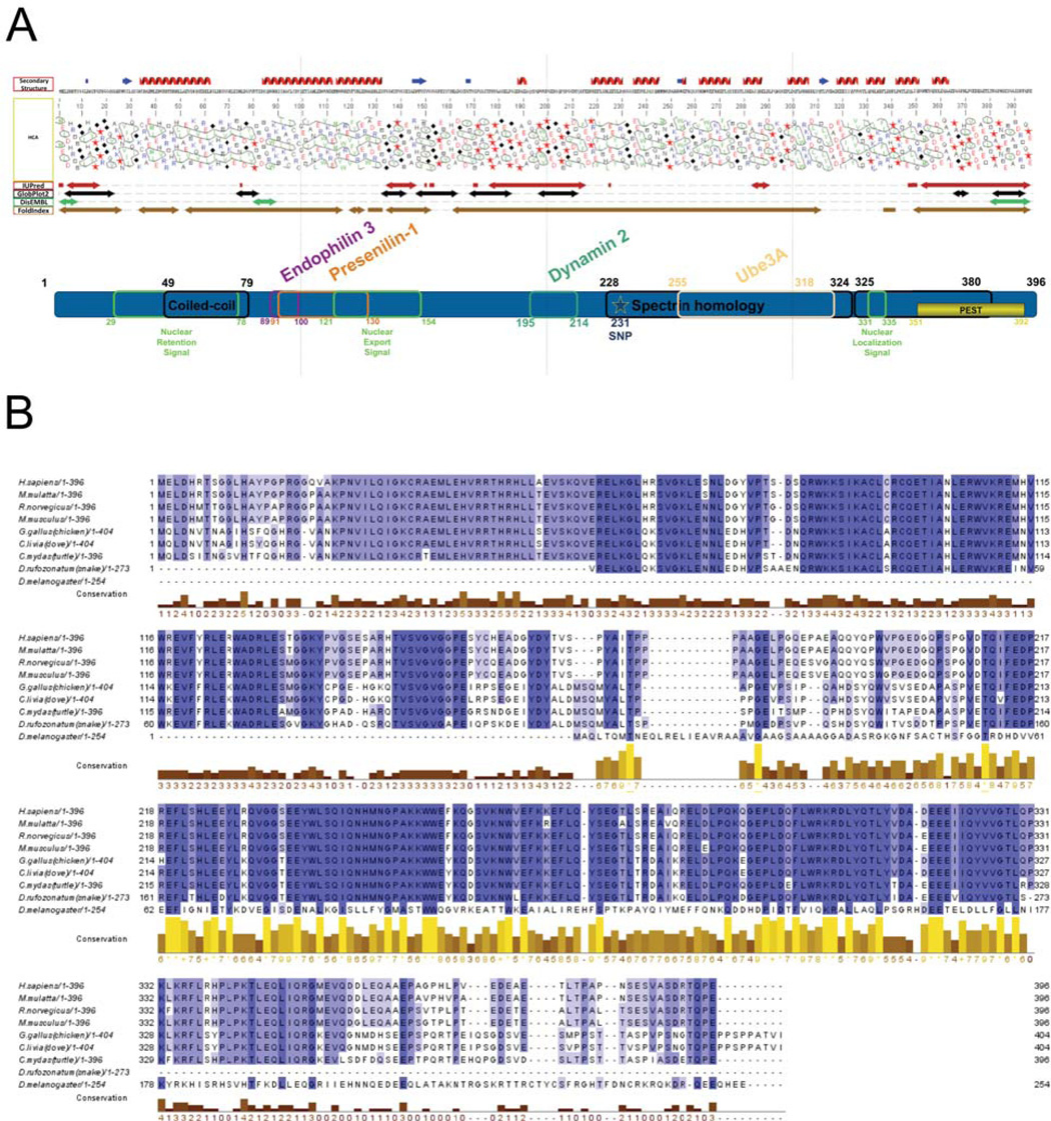
Schematic representation of hArc, with predicted secondary structure, functionally relevant regions and motifs, sequence alignment and domain organization (**A**) Prediction of secondary structure and disordered/ordered regions of hArc as estimated by MeDor [27]. At the topid, the predicted secondary structure. Below the primary sequence, an HCA plot shows the predicted regions of order and disorder. Symbols are used to represent residues with important structural characteristics [Proline (★), glycine (◆), threonine (■) and serine (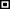)]. Four additional disorder predictors were used, namely IUPred [56], GlobPlot2 [57], DisEMBL [58] and FoldIndex [59]. Functionally significant regions of the Arc protein are shown below these predictions. The Arc regions involved in binding of four of the known partners are indicated on top whereas regions involved in localization are indicated at the bottom. (**B**) Multiple sequence alignment of Arc from various species was performed with Clustal Omega and displayed in Jalview. The conservation of residues is highlighted by shades of blue; the darker the colour, the more conserved the residue [26]. The extent of conservation was also calculated based on physicochemical properties of amino acid sets, indicated in the yellow/brown histogram.

To predict whether hArc is structured or contains disordered domains, MeDor, a meta-predictor of protein unstructured regions [27], was used to analyse the primary hArc sequence (Figure 1A). The HCA plot generated by MeDor predicted a central disordered region and smaller regions of disorder at the N- and C-termini. HCA also indicated two major domains containing mostly α-helices and a few β-strands between the regions of predicted disorder. Other disorder predictors were in large agreement with the HCA plot (Figure 1A, arrows below HCA plot). The multiple sequence alignment (Figure 1B), together with the order/disorder predictions (Figure 1A), revealed highly conserved C-terminal residues ~209–365 (≥87.6% sequence identity among sequences shown in Figure 1B), which correspond to the putative C-terminal domain. The smaller putative N-terminal domain (residues ~25—130) has a lower residue conservation (≥53.7% sequence identity; Figure 1B). Whereas this N-terminal domain is highly basic (computed isoelectric point (pI)=9.6), the larger C-terminal domain is acidic (computed pI=4.8). The full-length protein is slightly acidic (computed pI=5.5).

Predicted structural models of the putative N-terminal domain and the central linker region of hArc were obtained from MODBASE [29], which contains theoretically calculated models based on homology, whereas we modelled the C-terminal domain using the structure of two repeats of chicken α-spectrin as template (Supplementary Figure S1). Nevertheless, all structural templates for Arc show very low sequence identity (<35%) such that each of the domains and the resulting structural models are just moderately reliable.

### Preparation of recombinant hArc protein

To maximize the yield of soluble hArc protein, four different pET expression vectors were tested, each producing hArc with its N-terminal fused to a His-tagged partner protein. Ligation of the hArc open reading frame into pET MBP_1a, pET ZZ_1a, pET Trx_1a and pET GST_1a produces hArc with MBP, an IgG-binding ZZ-domain, Trx and GST respectively. Recombinant fusion proteins from all constructs were expressed in bacteria by auto-induction medium, allowing direct comparison of the expression level [31] (Supplementary Figure S2A). Since hArc expression was highest with the ZZ-tag, the auto-induction culture containing pET ZZ_1a–hArc was scaled up and hArc was purified as done in the expression screening (Supplementary Figures S2B and S2C). Similar yield and homogeneity was obtained for the V231G hArc variant. Immunoblot analysis confirmed that hArc was immunoreactive and the same size as endogenous hArc from human SH-SY5Y neuroblastoma cells (Supplementary Figure S2D).

### Limited proteolysis of purified hArc shows a protein with two domains

**Figure 2:**
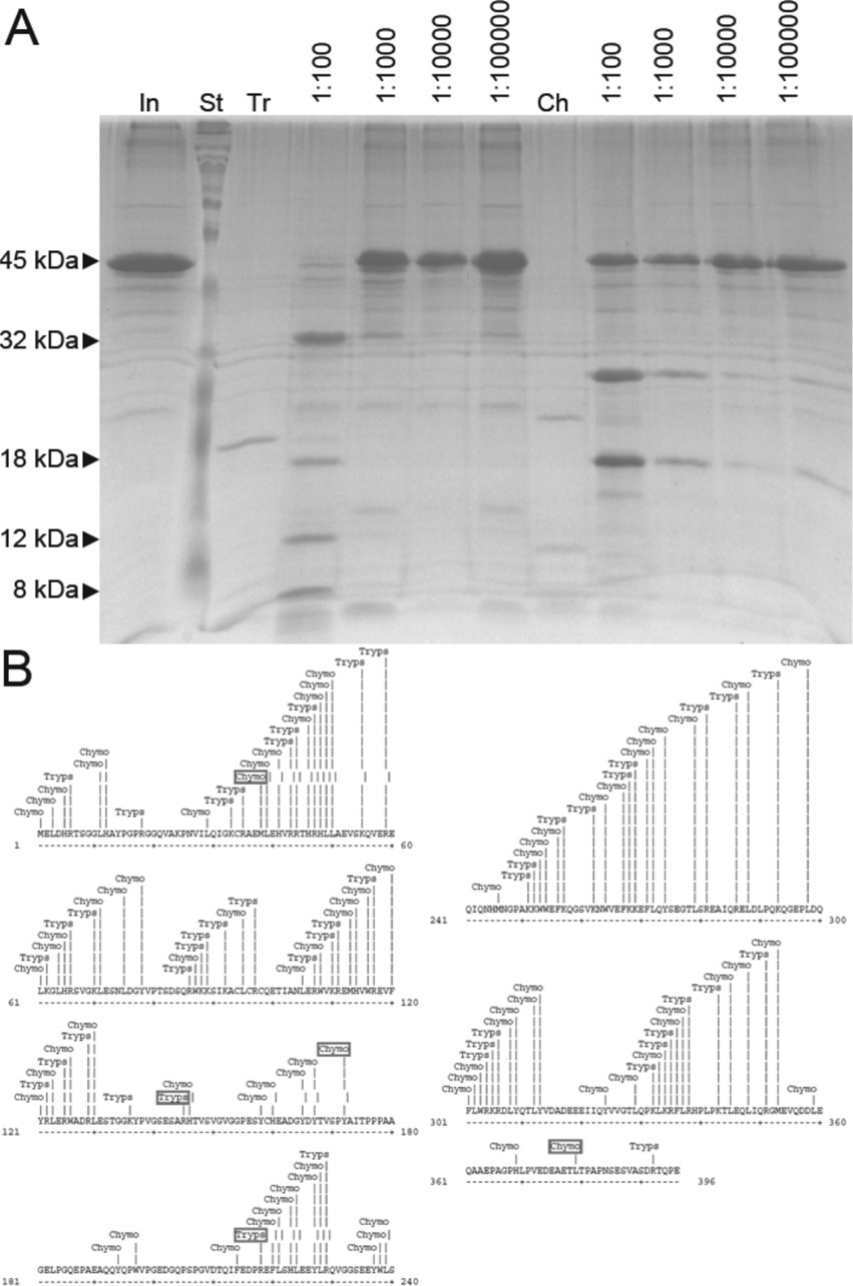
Limited proteolysis of hArc (**A**) Limited proteolysis was carried out with either trypsin (Tr) or chymotrypsin (Ch) at the indicated protease/hArc ratio. Trypsin resulted in four fragments (32, 18, 12 and 8 kDa) and chymotrypsin resulted in two major fragments (27 and 18 kDa) with a third 16-kDa band appearing after extended incubation periods. In, hArc input; St, standard. (**B**) Map of all potential hArc cleavage sites for chymotrypsin and trypsin. MS/MS-identified cleavage sites are indicated in boxes.

Limited proteolysis is often used in the identification of the boundaries of structural domains within multi-domain proteins and other proteins containing potentially unstructured regions. The basis for this relies on the increased sensitivity to protease digestion of unstructured regions. Under appropriate digestion conditions, well-structured domains typically accumulate as digestion products that can then be analysed by MS or other methods. Full-length hArc was subjected to limited proteolysis with trypsin and chymotrypsin and the reaction products were analysed by SDS/PAGE. Digestion of hArc with trypsin resulted in four fragments (32, 18, 12 and 8 kDa; Figure 2A) and chymotrypsin resulted in two major fragments (27 and 18 kDa; Figure 2A). A third 16-kDa band appeared after longer incubation times, which accumulated up to 80 min (time course not shown). The bands corresponding to the different degradation fragments were excised from the gels and subjected to MS analysis by LC–MS/MS after in-gel hydrolysis. The 27-kDa fragment produced by chymotrypsin digestion was identified as the region corresponding to hArc residues 173–379 (Figure 2B) with an expected molecular mass of 27.3 kDa. The 18-kDa fragment produced by chymotrypsin was identified as the N-terminal region including residues 1–172, with an expected molecular mass of 19.6 kDa, which is consistent with the apparent molecular mass estimated by SDS/PAGE. These fragments are consistent with a predicted chymotrypsin cleavage site at residue Tyr^172^ in the central region of hArc and are also in accordance with the apparent molecular mass seen by SDS/PAGE analysis. The 16-kDa band was not sufficiently abundant for MS analysis.

In agreement with these results, analysis of the trypsin digestion products indicated the presence of two highly accessible trypsin sites in the central region of the protein (residues 145 and 218; Figure 2B). These data indicate that full-length hArc is composed of two distinct N- and C-terminal domains separated by a highly protease-sensitive and, probably, less structured central region. Despite intense efforts, we were not able to purify isolated forms of the domains in the required amounts for biophysical and structural analyses.

### Secondary structure and conformational stability of hArc

**Figure 3:**
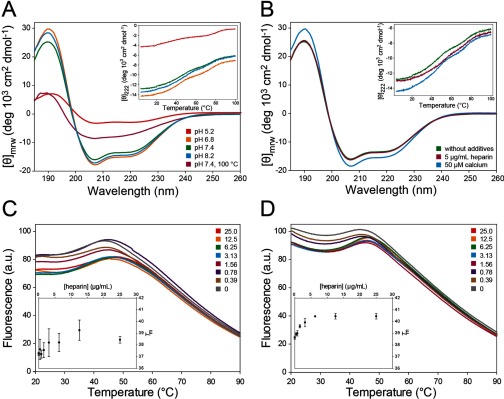
Far-UV CD and DSF analysis of hArc (**A** and **B**) CD spectra of hArc (4.4 μM) in 10 mM potassium phosphate at (**A**) pH 5.2 (red), 6.8 (orange), 7.4 (green) and 8.2 (blue) at 20°C and at pH 7.4 at 100°C (burgundy); CD spectrum taken at 20°C after heating hArc to 100°C is similar to that shown by the green line; and at (**B**) pH 7.4 (green), with 5 μg/ml heparin (burgundy) or 20 μM calcium (blue). Thermal scans are shown in insets. (**C** and **D**) DSF experiments where 2 μM hArc was incubated with the indicated concentrations of heparin (units are μg/ml) and subjected to a thermal gradient from 20°C to 90°C in 20 mM sodium Hepes, pH 7.0, without KF salt (**C**) and with 150 mM KF salt (**D**). The thermal denaturations monitored by SYPRO Orange fluorescence (*λ*_ex_=465 nm, *λ*_em_=610 nm) are shown in the main plots, whereas *T*_m_ values obtained from the maximum first derivative of the data are shown in the insets.

We analysed the secondary structure of recombinantly expressed hArc by far-UV CD spectra under various conditions (Figures 3A and 3B; Supplementary Figure S3). The CD spectrum of hArc at neutral pH revealed two local minima at 208 and 222 nm, which indicates a high α-helical content (Figure 3A, green line), in accordance with the prediction of secondary structure by MeDor (Figure 1) and by MODBASE (Supplementary Figure S1). The protein was stable in neutral–basic pH but showed a large loss of ellipticity at pH 5.2 (Figure 3A). In addition, remaining secondary structure was observed even after heating to 100°C (Figure 3A, burgundy line). This partial denaturation appeared fully reversible since the CD spectrum taken after cooling the sample to 20°C was similar to that obtained prior to heating (Figure 3A, green line). CDNN analysis [32] of the CD spectrum of hArc at pH 7.4 (20°C) predicted an α-helical and β-sheet structure of 42.1±0.3% and 12.8±0.2% respectively. The spectrum of the polymorphic variant V231G hArc, with a substitution in the proposed C-terminal domain, was very similar to that of WT hArc (Supplementary Figure S3A) with a slightly higher estimated α-helical content (51.0±0.4%).

We tested the effect of various ionic conditions and additions on the CD spectrum of hArc, such as calcium, the polyanion heparin and salt (KF). Heparin was added as a mimic of abundant polyanions in neurons, including the negatively charged plasma membrane, cytoskeletal proteins such as tubulin and actin, RNA and DNA [34]. These polyanions have shown chaperoning activity of interacting proteins *in vitro* [34]. None of these additives significantly altered the CD-estimated content of secondary structure of WT or V231G hArc variant (Figure 3B; Supplementary Figure S3B; and results not shown).

We then investigated hArc stability by differential scanning calorimetry (DSC), CD-monitored thermal denaturation and DSF (differential scanning fluorimetry) under various conditions. Whereas no distinct unfolding endotherm was observed by DSC (results not shown), which indicated a loose tertiary structure, thermal unfolding transitions were observed by both thermal CD (insets in Figures 3A and 3B; Supplementary Figures S3A and S3B) and DSF (Figures 3C and 3D). These melting transitions were, however, remarkably broad and non-co-operative, also pointing to a loose flexible tertiary structure. Thermal CD scans at pH 7.4 showed two overlapping unfolding events, with a *T*_m_ of 46.4±1.1°C for the first transition and 74.3±0.2°C for the second transition (Figure 3A inset; Table 1). The first transition was stabilized with heparin and slightly destabilized by increasing KF concentration, conditions that seem to exert opposite effects on the second transition (Figure 3B, inset; Table 1). A low concentration of calcium did not affect the corresponding *T*_m_-values (Figure 3B, inset; Table 1). Finally, V231G hArc also manifested two transitions (Supplementary Figure S3A, inset) and had similar *T*_m_ values to that of the WT protein.

**Table 1:** Midpoint denaturation temperatures (*T*_m1_ and *T*_m2_) for the melting transitions of hArc, measured by thermal-dependent CD and derivatization of the transitions hArc (4.4 μM) was prepared in 10 mM potassium phosphate at the indicated pH and conditions.

Condition	*T*_m1_ (°C)	*T*_m2_ (°C)
pH 6.3	43.2±0.9	75.0±0.6
pH 7.4	46.4±1.1	74.3±0.2
pH 8.2	48.5±0.1	73.8±1.0
pH 7.4, 150 mM KF	43.0±0.2	75.8±1.5
pH 7.4, 300 mM KF	42.7±0.4	77.6±0.1
pH 7.4, 5 μg/ml heparin	53.0±0.8	73.9±0.5
pH 7.4, 20 μM calcium	46.5±0.9	74.3±0.5

Further investigations on the conformational stability were performed by DSF, a sensitive technique that provides information on the thermal denaturation transitions of proteins and detects stabilizing ligand interactions [35,36]. Remarkably, only the first, low *T*_m_ transition was detected by DSF, whereas the second appears to be lost due to baseline decay and distortion associated with aggregation of the protein following the unfolding of the first domain (Figures 3C and 3D) [37]. The *T*_m_ value obtained for this transition at pH 7.4 without salt (*T*_m_=37.2±0.1°C) or with 150 mM KF (*T*_m_=38.6±0.1°C) was lower than those obtained by thermal CD, notably in the absence of salt (Table 1). Nonetheless, DSF experiments strongly supported the stabilization of this first unfolding transition of hArc by heparin, both in the absence (Figure 3C) and in the presence of 150 mM KF salt (Figure 3D).

In agreement with the domain organization predicted from the sequence analyses and alignments (Figure 1), the CD results show an hArc protein with high amounts of α-helical structure and indicate the presence of a modular organization with two major domains and a loose, flexible tertiary structure. The more stable domain (*T*_m_ about 74°C at pH 7.4) is further stabilized by KF and the domain with the lower *T*_m_ binds to and is stabilized by heparin (Table 1), as also corroborated by DSF. Based on the size of the domains (N-terminal residues 25–130, C-terminal residues 209–365) as well as the distribution and balance of charged residues [38], we predicted that the first and second thermal transitions corresponded to the N-terminal and C-terminal domains respectively. The fact that the N-terminal domain of hArc (residues ~25–130) was highly basic (pI=9.6) further corroborated this prediction, explaining its interaction with and stabilization by heparin. Interestingly, the N-terminal domain showed a high homology with human Janus kinase and microtubule-interacting protein 1, also called Marlin-1 (27% sequence identity between residues 56–106 of hArc and 325–375 of Marlin-1), with a remarkable co-distribution of positively charged residues (Supplementary Figure S4). On the other hand, our DSF results suggested a possible aggregation tendency of the larger and more stable C-terminal domain and we investigated this feature in more detail.

### Oligomeric distribution of hArc

**Figure 4:**
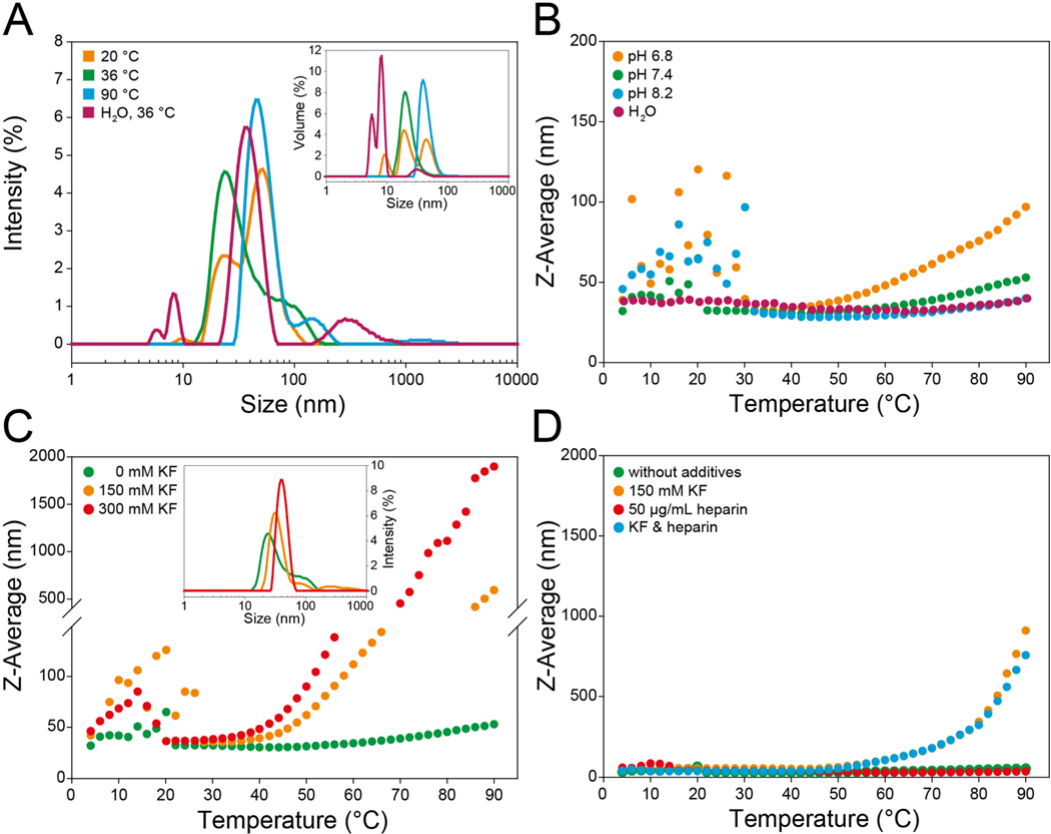
Size distribution of hArc studied by DLS hArc was prepared under various conditions at 22 μM. (**A**) Intensity size distribution of hArc in 10 mM potassium phosphate, pH 7.4, at 20°C (orange), 36°C (green) and 90°C (blue) and in distilled water at 36°C (burgundy). Inset: volume size distribution of hArc at corresponding temperatures. (**B**) Thermal scans of hArc in 10 mM potassium phosphate at pH 6.8 (orange), pH 7.4 (green) and pH 8.2 (blue) and in distilled water (burgundy). (**C**) Effect of increasing temperature on Z-average diameter of hArc in 10 mM potassium phosphate, pH 7.4, without added salt (green), with 150 mM KF (orange) and 300 mM KF (red). Inset: intensity size distribution of hArc under the same conditions at 36°C. (**D**) Effect of temperature on Z-average diameter of hArc in 10 mM potassium phosphate, pH 7.4 (green) or in the same buffer with either 150 mM KF (orange) or 50 μg/ml heparin (red) or both KF and heparin at the same concentrations (blue).

To further investigate the homogeneity and oligomeric state of recombinant hArc under various conditions, DLS was carried out. The plots for intensity size distribution revealed a unique population with a hydrodynamic diameter (*D*_h_) of 33.9±8.0 nm (22.6±0.6 nm for volume size distribution) in 10 mM potassium phosphate buffer, pH 7.4, at 36°C. This size corresponds to an estimated molecular mass of >552 kDa for a protein with some elongation, indicating that under these conditions hArc is an oligomer with >12 subunits. Populations with larger sizes (approximately *D*_h_=45 nm) corresponding to large clusters of self-associated hArc (≳40 subunits) were also observed at higher and lower temperatures (90°C and 20°C respectively; Figure 4A). As better seen in the volume size distribution (Figure 4A, inset), another population of a smaller diameter (about 8.1–9.6 nm) was also observed at 20°C in buffer without salt. Moreover, when hArc was prepared in double-distilled water, a smaller size population was also observed (*D*_h_=5.7 nm), corresponding to an estimated molecular mass of 48 kDa, i.e. monomeric hArc (Figure 4A, inset). Thus, the species with *D*_h_=8.1–9.4 nm, appearing in both water and buffer without salt might be either dimers or monomeric forms of hArc with a very elongated shape. The propensity to form large oligomers/aggregates (diameter >43.8 nm) in a temperature-dependent manner (notably at temperatures > 40°C) increased with decreasing pH (Figure 4B) and with increasing salt content (Figure 4C). The V231G hArc polymorphism or the addition of calcium (results not shown) or heparin (Figure 4D) did not affect the size distribution or thermal-dependent aggregation propensity. Furthermore, the increased propensity for oligomerization/aggregation in the presence of salt was observed both with and without heparin (Figure 4D). The hArc populations of different sizes were interconverted by changing the buffer conditions. Oligomerization thus appeared to be reversible. The experiments shown in Figure 4 were all performed at a concentration of hArc of 1 mg/ml (22 μM subunit) and, as expected from the Law of Mass Action, higher concentrations exacerbated the oligomerization/aggregation of the protein (results not shown).

A particular feature revealed by the DLS-monitored thermal scans, under all conditions, was that recombinant hArc undergoes oligomerization in the 4–30°C range, which was reversible since the thermal DLS was completed in the same sample in the 4–90°C range (Figures 4B and 4C).

### Visualization of hArc by EM and AFM

**Figure 5:**
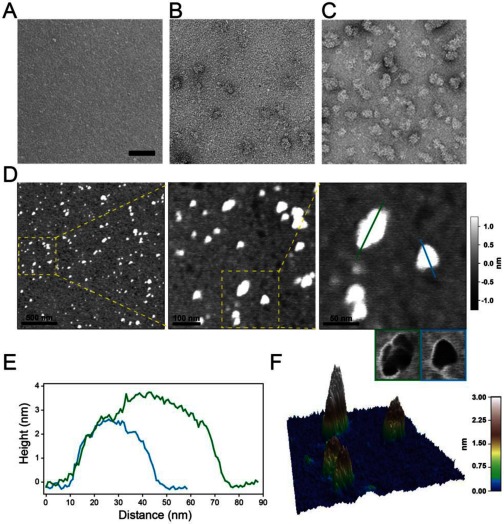
Visualization of hArc by EM and AFM (**A**–**C**) EM analyses of hArc. Samples of hArc (15 μg/ml; 0.33 μM) were prepared in water (**A**), in 20 mM sodium Hepes, pH 7.4 (**B**) and in the same buffer with 125 mM KCl (**C**). (**D**–**F**) AFM images of hArc. Samples were prepared in 10 mM potassium phosphate, pH 7.4, diffused on a mica surface. (**D**) Acquired height images of 2 μm, 500 nm and 200 nm scan sizes (from left to right). The rectangles mark the close-up of the following panel. In the right close-up panel, small images with blue and green framing present the phase image of the protein shown in the height image above. (**E**) Cross-section of the blue and green line in (**D**). (**F**) 3D representation of the 200 nm scan size image shown in (**D**).

As indicated by DLS experiments, hArc in water and a major fraction of hArc in buffer of low ionic strength is monomeric. Thus, given its low molecular mass, hArc in water could not be visualized with the electron microscope (Figure 5A). Heparin, present at a final concentration up to 0.5 mg/ml, did not modify the monomeric state (results not shown). However, when the aqueous solution contained 20 mM sodium Hepes buffer, sparse aggregation occurred (Figure 5B). The tendency of hArc to aggregate in salt solution was readily observed by EM in buffer with 125 mM KCl (Figure 5C).

Further visualization of the structural and oligomeric state of recombinant hArc was performed by AFM (Figures 5D–5F). The protein was deposited on a freshly cleaved mica surface, dried and imaged. The images confirm that in buffer without salt at pH 7.4, a combination of monodisperse and oliogomeric distribution of hArc was obtained (Figures 5D–5F). We note that the diameter-to-height profile of a protein imaged in a non-aqueous environment may be subject to deformation and flattening by the cantilever tip (Figure 5E). Nevertheless, the AFM images clearly showed hArc to be pyramidal in shape in the monomeric state (Figures 5D–5F).

### Binding of a presenilin-1 peptide

**Figure 6:**
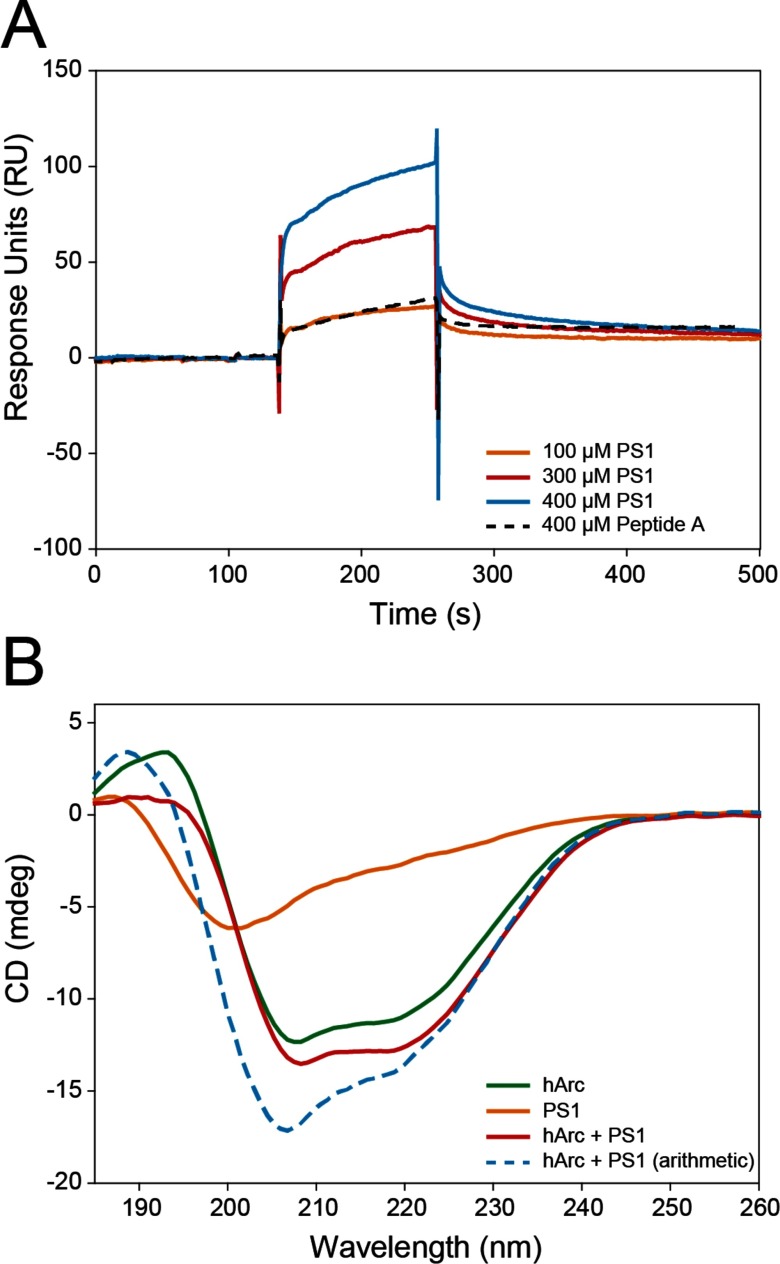
Interaction of PS1 with hArc (**A**) SPR sensorgrams showing binding of the PS1 peptide (unbroken lines) to hArc with increasing peptide concentrations (up to 400 μM) at 25°C in HBS-EP, pH 7.4, buffer. A *K*_d_ value of 42±8 μM was obtained from the analysis of the sensorgrams by fitting a 1:1 Langmuir binding model. Peptide A (dashed line showing result at 400 μM) was used as a negative control and showed very weak binding. (**B**) Far-UV CD spectra of hArc (green), PS1 (orange) and hArc together with PS1 (red). The concentrations were 4 μM for hArc and 10 μM for PS1 and samples were prepared in 10 mM potassium phosphate, pH 7.4. The CD spectra were taken at 20°C and are buffer-subtracted. The blue dashed line represents the arithmetic addition of the CD spectra for hArc and PS1 alone.

We also sought to confirm the functionality of recombinant hArc by its ability to associate with PS1, a known Arc binding partner. PS1 binds through its N-terminal region (residues 1–40) within amino acid region 91–130 of hArc (Figure 1A) [14]. We investigated the binding of the N-terminal region of PS1 to recombinant hArc by SPR and CD (Figure 6). As seen by SPR analyses, PS1 interacts with hArc. The sensorgrams for PS1 binding at increasing peptide concentration are shown in Figure 6A. The binding was concentration-dependent and resulted in a *K*_d_ value of 42±8 μM. Furthermore, as seen by comparing the CD spectra for hArc and PS1, both separate and together, the binding also induced a conformational change in the components of the hArc–PS1 complex (Figure 6B).

## DISCUSSION

### Secondary and tertiary structure of hArc

**Figure 7:**
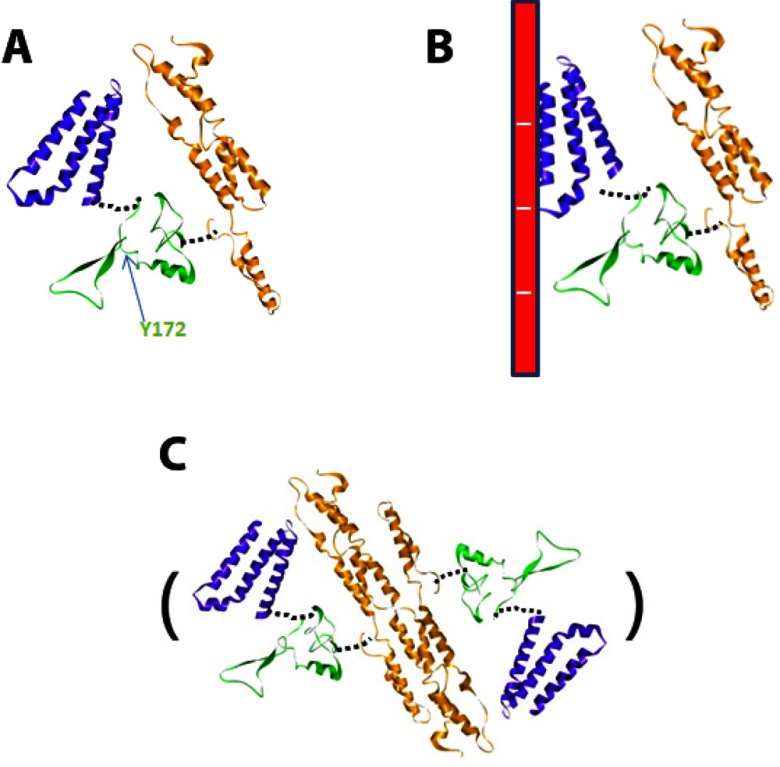
Putative modelled structure of hArc under different conditions The structure of the basic N-terminal domain (blue), the middle linker region (green) and the C-terminal domain (orange) corresponds to that of the models in Supplementary Figure S1. The monomeric hArc at low ionic strength (**A**) and interacting with polyanions through the N-terminal domain (**B**). (**C**) Dimeric hArc, formed through dimerization of the C-terminal domain. Further tetramerization and oligomerization of hArc might occur following a similar arrangement of these dimeric units as for spectrin [60].

In the present work, we propose a model for hArc as a protein containing two domains with a large content of helical structure on either side of a central, more disordered linker region (Figure 7). A major chymotryptic cleavage site (Tyr^172^) is located in the linker region (Figure 7A). CD analyses confirmed high α-helical content in hArc (~42%), whereas thermal denaturation analyses displayed non-co-operative melting indicating independent structural domains, each of which unfolds at a different temperature (*T*_m_ values of ~46°C and ~74°C, pH 7.4). Additional information on the thermal stability of the N-terminal domain was obtained by DSF, which provided lower *T*_m_ values than those obtained with thermal-dependent CD, indicating that the loose 3D structure of hArc is less stable than the secondary structure.

Several characteristics aided in the assignment of each thermal transition to the respective N- and C-terminal domains. The putative C-terminal domain is exceptionally well-conserved (residues ~209–365; ≥87.6% sequence identity; Figure 1B). We therefore posit that this is the very stable domain (*T*_m_ ~74°C) since evolutionary mutations, which favour protein stability, are better conserved [39]. On the other hand, the N-terminal domain (residues ~25–130; ≥53.7% sequence identity), with a predicted unbalanced charge distribution at neutral pH (pI=9.6), shows a lower stability (*T*_m_ ~46°C) and a concomitant stabilization by higher pH. The interaction of this domain with the polyanion heparin further supports these domain assignments and points to a possible physiological significance of the interaction of hArc with polyanions [40]. The interesting sequence identity with Marlin-1, a microtubule-interacting protein implicated in protein trafficking in neuronal dendrites [41,42], further supports that the basic N-terminal domain of Arc may interact with polyanionic cytoskeletal components (Figure 7B) *in vivo*. Arc has been shown to co-sediment with F-actin but does not bind to monomeric actin [7]. During LTP in the dentate gyrus, Arc protein synthesis is required to stabilize expansion of the F-actin network at synapses [17]. However, interactions between Arc and the actin cytoskeleton are little understood. On the other hand, Arc has also been suggested to interact with newly polymerized microtubules [22]. Microtubule incursions into dendritic spines and the coupling of microtubules to spine F-actin are important events in the structural plasticity of dendritic spines [43,44].

Despite the high amount of observed (Figures 3A and 3B; Supplementary Figures S3A and S3B) and predicted (Figure 1A; Supplementary Figure S1) α-helical secondary structure in hArc, hArc appears to be a flexible and modular protein, with loose 3D structure both within each domain and between domains (Figure 7A). Thus, the thermal transitions are separated and non-co-operative. The N-terminal domain is stabilized by heparin binding as seen by the increased *T*_m_ for this domain, whereas salt stabilizes the C-terminal domain (Table 1; also see the next section), compatible with oligomerization. Furthermore, stabilization of one domain leads to a concomitant decrease in the corresponding *T*_m_ for the other domain, as measured by thermal-dependent CD (Table 1), suggesting that the domains separate upon their respective interactions (Figures 7B and 7C).

### Monomeric/oligomeric hArc

Our biophysical investigations, EM micrographs and AFM images led us to a monomeric model of hArc. The monomeric species is favoured at low ionic strength, with a modular disposition of the domains and scarce inter-domain interactions, resulting in a pyramid-shaped protein (Figures 5D–5F; see putative model in Figure 7A). The effect of salt on the stability of the protein was remarkable. Salt increases the aggregation propensity of hArc and it does so with a concomitant stabilization of the C-terminal domain. Given the homology of the C-terminal domain with α-spectrin repeats 21 and 22 [7,8] and the known propensity of spectrin structural motifs to form dimers and oligomers [45], we propose that the hArc C-terminal domain drives oligomerization (Figure 7C). Dimerization of coiled-coil helical motifs of spectrin causes a substantial stabilization of its structure, which may be imitated in hArc oligomerization dynamics.

The reversible tendency of hArc to oligomerize at low temperatures (~4°C–30°C) illustrates the hydrophobic effect, which seems to be crucial to both stabilize the C-terminal domain and increase intermolecular interactions that could account for hArc oligomerization, as described in other systems [46]. hArc probably displays a propensity to aggregate at a temperature range where most mesophilic proteins have maximum stability, around 17°C [47].

### Physiological consequences of Arc as a protein capable of reversible self-association

Arc is able to interact with multiple protein partners and is implicated in distinct neuronal mechanisms and forms of synaptic plasticity. Its interaction with endophilin-3 and dynamin-2 promotes internalization of AMPA-type glutamate receptors [12]. During periods of synaptic inactivity, calcium/calmodulin-dependent protein kinase II-β serves as an anchor for Arc in dendritic spines, thereby ‘tagging’ specific synaptic populations for the Arc-dependent clearance of AMPA-type glutamate receptors [48]. The Arc partner PS1 is a core component of the γ-secretase complex, which cleaves APP to generate the pathological amyloid β-peptides(Aβs) in Alzheimer's disease. Arc interaction with PS1 causes an increase in APP endocytosis, thereby promoting Aβ formation and accumulation [14]. In the present study, we find that binding of PS1 peptide results in structural changes of the peptide–protein complex, as measured by CD and SPR. PS1 binds at the interphase between the N-terminal domain and the disordered central region, whereas dynamin-2 binds Arc residues 195–214 [12], at the interphase between the central region and the second domain. Highly-connected hub proteins have been shown to have more observed and predicted disordered regions [49]. Disordered regions can enhance protein turnover in the absence of a ligand [50], increase the speed of interactions [51] and enhance overall flexibility and folding [52]. A flexible linker region between the two elongated hArc domains may allow them to move freely with respect to each other and confer the recognition of, and binding to, multiple targets. This disordered region may therefore be central in regulating Arc protein–protein interactions.

Evidence suggests that hArc is a flexible hub protein capable of interacting with diverse protein effectors. Hub proteins such as hArc have been reported to play essential roles in cellular regulation and tend to be highly conserved across species [53]. AFM and EM images of hArc show both monomeric and oligomeric formations pointing to possible coexistence of multiple pools of Arc in the cell, with distinct functional capabilities and rates of degradation. Self-oligomerization of hArc may serve as a hub to spatially concentrate protein partners within neuronal sub-compartments (e.g. dendritic spines or nuclear domains). During LTP consolidation in the dentate gyrus, Arc synthesis serves to promote cofilin phosphorylation and actin cytoskeletal reorganization at synapses. This appears to be a highly dynamic process dependent on the sustained rapid synthesis and degradation of Arc protein [17,19,54,55]. As oligomers are expected to have slow turnover, it is likely that the fast actions of Arc of the order of minutes depend on the monomeric form. However, further work is needed to more clearly identify and characterize the precise roles of monomeric and oligomeric Arc protein.

### The polymorphic variant V231G hArc

A single polymorphism can have profound effects on protein structure and/or function. Only one known missense variation occurs in the hArc protein (rs201562490; V231G) and it has previously not been characterized. We analysed the V231G polymorphism with regard to structure and stability and observed only slight changes in secondary structure content for V231G hArc. This particular variation occurs in 2–4.6% of the population, so a profound deleterious or even beneficial phenotype is unlikely as it would have been uncovered. Thus, the lack of dramatic changes in stability is as expected. However, further studies are necessary to investigate whether the polymorphism whether alters the binding affinity of various partners and/or regulates Arc's response to various cytosolic environments.

In conclusion, the present study elucidates basic physicochemical and structural properties of hArc. The findings are important for the elucidation of the molecular function of Arc as a master regulator of activity-dependent synaptic plasticity and brain adaptive mechanisms, including memory formation. Moreover, the work paves the way for in-depth crystallographic analysis of the hArc protein structure.

## Online data

Supplementary data
