# Supplementary material for: Arc is a flexible modular protein capable of reversible self-oligomerization
Source: Biochem J. 2015 May 5;468(Pt 1):145–58. doi: 10.1042/BJ20141446 (PMC4422259; doi:10.1042/BJ20141446)
Supplement: Supplementary data [file bj4680145ntsadd.pdf]

Supplemental information

Figure S1. Theoretical homology-based structural models of the two putative Arc domains and a central disordered region.

Models of the N-terminal domain (residues 54–137) and middle region (residues 140–220) were obtained from MODBASE (Pieper et al., 2011); the templates were residues 45–126 of protein TM1646 from *Thermotoga maritime* (PDB ID: 2P61; 24% sequence identity) and 48–149 of anti-ECF sigma factor, ChrR, from *Marinobacter hydrocarbonoclasticus VT8* (PDB ID 3O14; 32% sequence identity), respectively. The C-terminal domain, residues 216–378, was prepared using SWISS-MODEL [61], with two repeats of chicken spectrin (residues 1813–1982; PDB ID: 1CUN) as template (26.2% sequence identity).

Figure S2. Expression and purification of hArc.

(**A-C**) SDS-PAGE gels showing fractions from the purification. (**A**) hArc was expressed and purified with different N-terminal fusion partners: thioredoxin (Trx; 14 kDa), glutathione S-transferase (GST; 26 kDa), maltose-binding protein (MBP; 45 kDa), all of which were cleaved off by TEV (25 kDa). SDS-PAGE gel showing the cleavage of the four different fusion proteins at a ratio of 1 µg:100 µg (TEV:fusion protein) for 12 h at 4 °C, leaving hArc (50 kDa) separated from the partners. (**B**) The lysate (L) was centrifuged and the supernatant (SN) was applied to Ni-NTA resin. The column was washed with multiple buffers (W1-4) and the bound protein eluted (E). (**C**) The His_6-ZZ-hARC was digested with TEV protease (D), reloaded on a Ni-NTA column, and purified hArc protein (PP) was collected in the flow-through. (**D**) Purified protein (PP) was analyzed by western blot with Arc C-7 antibody and compared to endogenous (End.) Arc in human SH-SY5Y cells.

Figure S3. **CD analysis of hArc.**

Far-UV CD spectra at 20 °C and thermal scans (insets) of (**A**) 4.4 µM hArc (green) and V231G-hArc (blue) in 10 mM K-phosphate, pH 7.4, and of (**B**) of 4.4 µM hArc in 10 mM K-phosphate, pH 7.4 (green), with addition of 150 mM KF (orange) or 300 mM KF (red).

Figure S4. Sequence similarity between the N-terminal domain of hArc and the microtubule-binding region of Marlin-1.

Sequence alignment of the N-terminal region of hArc (Q7LC44; residues 56–106) and residues 325–375 of Janus kinase and microtubule-interacting protein 1, also called Marlin-1 (Q96N16-7), showing 27% sequence identity, and a high co-distribution of positively-charged residues (marked in green).

Supplementary Figure S1

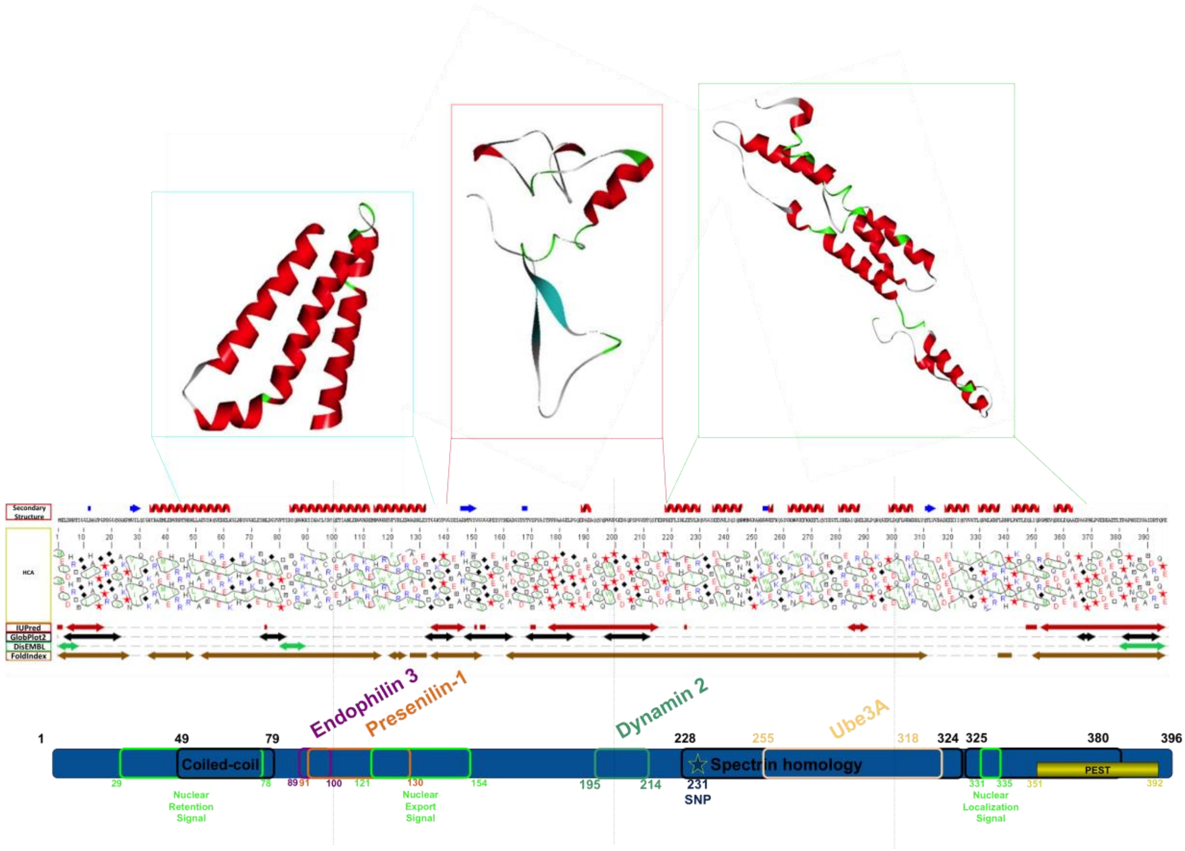

Supplementary Figure S2

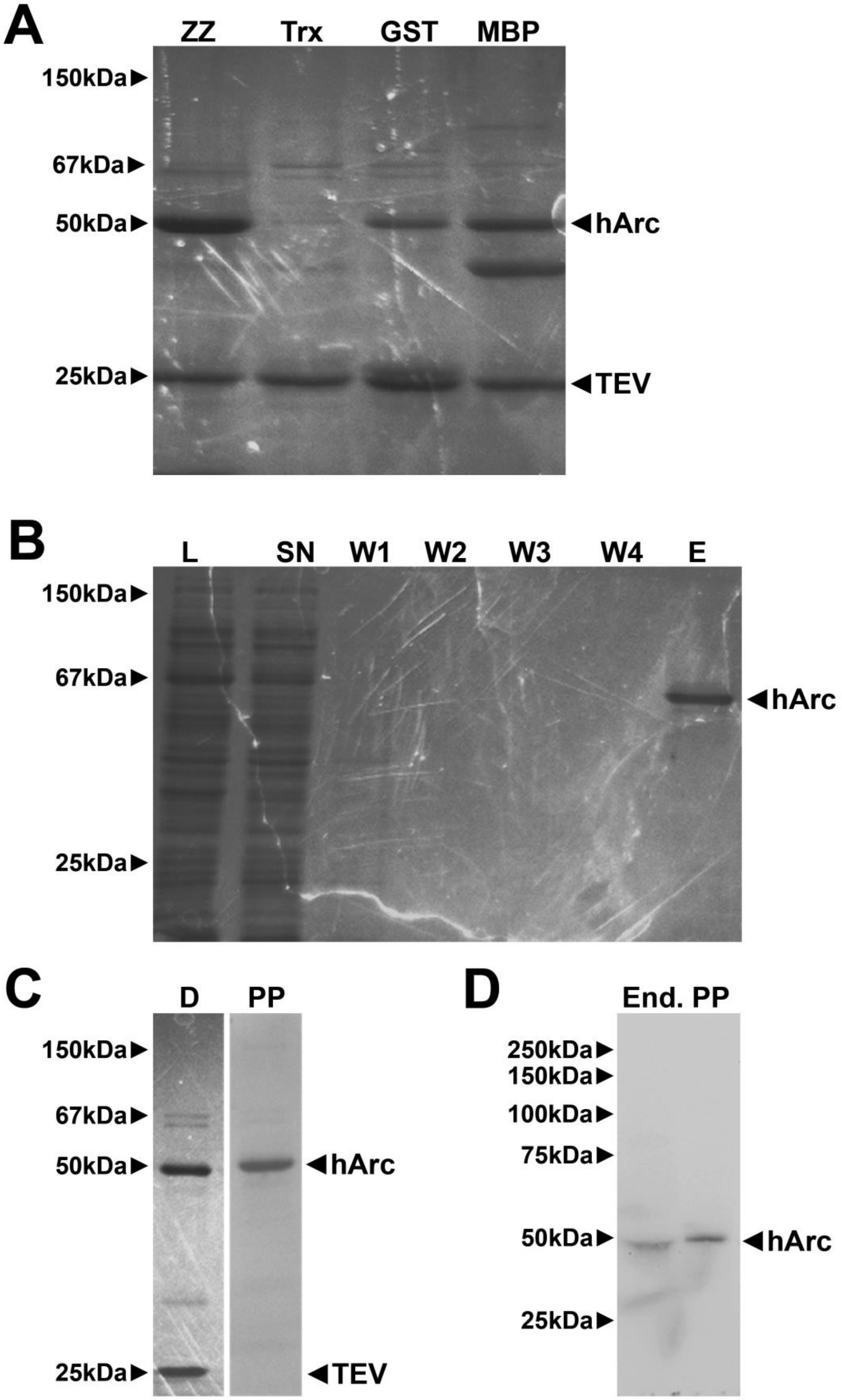

Supplementary Figure S3

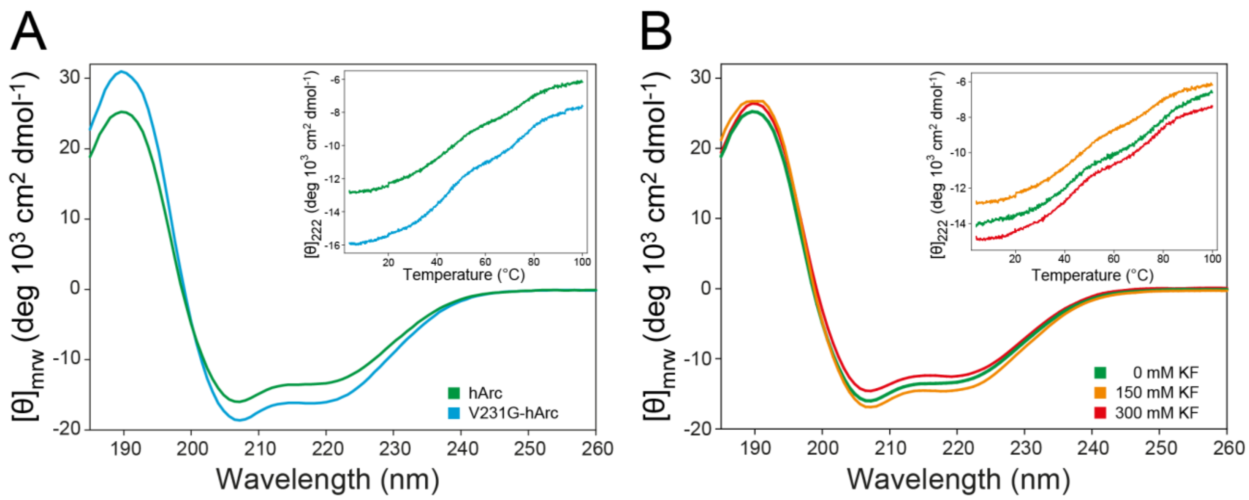

Supplementary Figure S4

```
56    QVERELKGLHRSVGKLESNLDGYVPTSDSQRWKKSIKACLCRCQETIANLE---------   106 Q7LC44
      Q+ RE + L R+   L+  + G +      R ++ ++A L RCQ   I +LE
325   QLTREYQALQRAYALLQEQVGGTLDAEREARTREQLQADLLRCQAKIEDLEKLLVEKGQD   384 Q96N16-7

107   -RWVKREMHVWR------EVFYRLERWADRLES   132 Q7LC44
       +WV+ +  + R      E  YRLE   ++L++
385   SKWVEEKQLLIRTNQDLLEKIYRLEMEENQLKN   417 Q96N16-7
```
